# Exploring the Behavior and Metabolic Transformations of SeNPs in Exposed Lactic Acid Bacteria. Effect of Nanoparticles Coating Agent

**DOI:** 10.3390/ijms18081712

**Published:** 2017-08-05

**Authors:** Maria Palomo-Siguero, Yolanda Madrid

**Affiliations:** Departamento de Quimica Analitica, Facultad de Ciencias Quimicas, Universidad Complutense de Madrid, Avda Complutense s/n, 28040 Madrid, Spain; mpsiguero@ucm.es

**Keywords:** selenium nanoparticles, lactic acid bacteria, metabolism, inductively coupled plasma mass spectrometry, flow cytometry, transmission electron microscopy

## Abstract

The behavior and transformation of selenium nanoparticles (SeNPs) in living systems such as microorganisms is largely unknown. To address this knowledge gap, we examined the effect of three types of SeNP suspensions toward *Lactobacillus delbrueckii* subsp. *bulgaricus* LB-12 using a variety of techniques. SeNPs were synthesized using three types of coating agents (chitosan (CS-SeNPs), hydroxyethyl cellulose (HEC-SeNPs) and a non-ionic surfactant, surfynol (ethoxylated-SeNPs)). Morphologies of SeNPs were all spherical. Transmission electron microscopy (TEM) was used to locate SeNPs in the bacteria. High performance liquid chromatography (HPLC) on line coupled to inductively coupled plasma mass spectrometry (ICP-MS) was applied to evaluate SeNP transformation by bacteria. Finally, flow cytometry employing the live/dead test and optical density measurements at 600 nm (OD_600_) were used for evaluating the percentages of bacteria viability when supplementing with SeNPs. Negligible damage was detected by flow cytometry when bacteria were exposed to HEC-SeNPs or CS-SeNPs at a level of 10 μg Se mL^−1^. In contrast, ethoxylated-SeNPs were found to be the most harmful nanoparticles toward bacteria. CS-SeNPs passed through the membrane without causing damage. Once inside, SeNPs were metabolically transformed to organic selenium compounds. Results evidenced the importance of capping agents when establishing the true behavior of NPs.

## 1. Introduction

Nanomaterials (within 1 nm to 100 nm size) are currently employed in many fields such as science, technology and even consumer products. The increasing use of nanoparticles (NPs) requires a proper assessment of their impact on environmental and human health. To date, the potential toxicity of nanoparticles and their interaction mechanisms with cells and living organisms has not been fully assessed. A huge number of studies are associated with elucidating the toxicity mechanisms of NPs. The proposed mechanisms include: interaction of NPs with cell membranes producing physical damage, NP internalization resulting in cell malfunction, production of reactive oxygen species, and inhibition of protein function [1,2]. NP toxicity is affected by parameters such as, size, morphology, chemical composition and the nature of the stabilizer. It is accepted that the fate and toxicity of NPs are largely influenced by the physical interaction between the NP surface and the cellular membranes or bacteria examined. Several authors even indicate that the capping agent located on the surface to enhance stability is the most important factor in evaluating NP toxicity [3]. Therefore, the use of stabilizers may hinder the normal utilization of synthesized nanoparticles in biological applications since their chemical nature may be toxic. These data suggest that size alone is not the exclusive determining toxicity factor. Moreover, NPs will be subjected to dynamic physical and chemical conditions which result in transformation to different end-products.

Selenium (Se) is considered an essential element for human health. The essentiality of selenium is related to its presence in selenoproteins as selenocysteine [4]. Beneficial effects of Se have been found to be dependent on the supplemental form and its efficacy. Selenium nanoparticles (SeNPs) have been lately considered as a new chemical form of selenium. SeNPs have attracted the attention of many researchers due to their attractive features. For instance, SeNPs appear to be very effective as antioxidant agent, due to their capability of producing antioxidant selenoproteins such as glutathione peroxidise (GPx) and thioredoxin reductase (TRx) [5,6]. Others reported the antimicrobial effect of SeNPs [7]. For all mentioned, the applications of SeNPs has increased in importance, especially in the medical and clinical field [8], in electronics and sensors development [9] and in food packaging applications [10].

The main synthetic approach for preparing SeNPs is by chemical reduction, employing a reducing agent (citric and ascorbic acids and gluthatione, for instance) and stabilizers such as proteins such as bovine serum albumine (BSA) or water-soluble polysaccharides [11]. Biogenic synthesis of SeNPs has gained acceptance in the last few years due to its simplicity, low cost, and biocompatibility of the resulting nanostructures with biomedical applications. Several papers have appeared in the literature describing the microbial synthesis of SeNPs [12,13,14,15]. In these studies, the role of biogenic organic compounds such as proteins, lipids and polysaccharides that act as capping agents are highlighted. These biogenic capping agents control SeNPs size, favor SeNPs stabilization in aqueous solution due to the presence of negatively charged functional groups, change the properties of SeNPs, and consequently their impact on environment and human health.

Very little data on the effect of SeNPs on lactic acid bacteria has been published. The accumulation and biotransformation of selenium by lactic bacteria has appeared in several papers [16,17,18,19,20]. These studies evidence that selenium, once accumulated by *Lactobacillus* species is biotransformed into selenoamino acids such as selenomethionine (SeMet) and selenocysteine (SeCys) However, in all above-mentioned studies, selenium was supplemented as inorganic selenium.

The main goal of the current work was focused on evaluating the effect of three different SeNPs suspensions on *Lactobacillus delbrueckii* subsp. *bulgaricus* LB-12 (Gram-positive bacteria). For achieving this goal, a multi-technique platform was employed. Transmission electron microscopy (TEM) was used to locate SeNPs in the bacteria. High performance liquid chromatography (HPLC) on line coupled to inductively coupled plasma mass spectrometry (ICP-MS) was applied to evaluate SeNPs transformation by bacteria. Finally, flow cytometry employing the live/dead test and optical density measurements at 600 nm (OD_600_) were used for evaluating the percentages of bacteria viability when supplementing with SeNPs. SeNP synthesis was performed in presence of a polysaccharide, [e.g., chitosan, a Poly(d-glucosamine)]; a polymer [hydroxyethyl cellulose (HEC)] and an ethoxylated non-ionic surfactant [2,4,7,9-tetramethyl-5-decyne-4,7-diol ethoxylate] as capping agents.

## 2. Results and Discussion

### 2.1. Behaviour of Selenium Nanoparticles (SeNPs) in Exposed L. d. Bulgaricus LB-12

**Figure 1:**
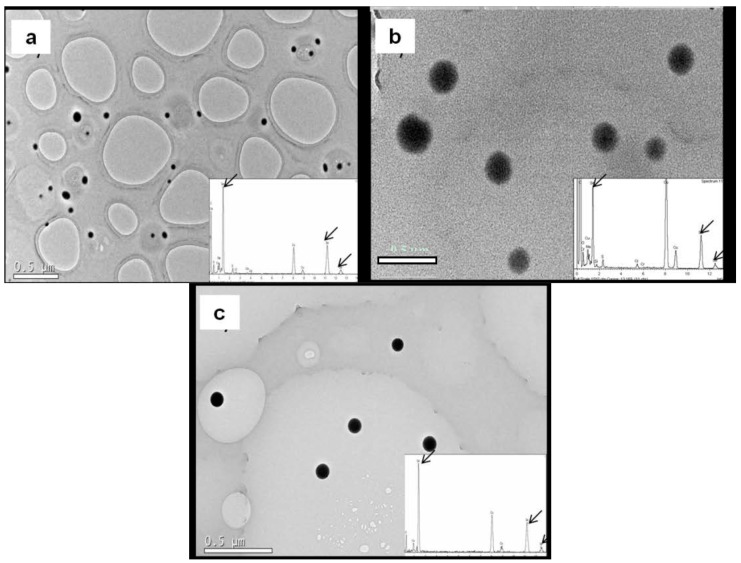
Transmission electron microscopy (TEM) images and X-ray energy dispersive spectroscopy (XEDS) spectrum of chitosan selenium nanoparticles (CS-SeNPs) (56 ± 5 nm) (**a**), Ethoxilated-SeNPs (53 ± 6 nm) (**b**) and hydroxyethyl cellulose (HEC)-SeNPs (60 ± 6 nm) (**c**) at pH = 5, *T* = 20 ± 1 °C. Black arrows indicate the Se emission peaks consisting of SeL_α_, SeK_α_ and SeK_β_ at 1.4, 11.22 and 12.49 keV, respectively.

Synthesis of SeNPs was carried out by applying a chemical process developed by Bai et al. [11] that is based on the reduction of selenite with ascorbic acid in presence of different stabilizers agents (Chitosan, 2,4,7,9-tetramethyl-5-decyne-4,7-diol ethoxylated (named as Surfynol) and Hydroxyethyl cellulose). As shown in Figure 1, the morphologies of the resulting SeNPs were all spherical, with diameters of 56 ± 5 nm for chitosan selenium nanoparticles (CS-SeNPs) (Figure 1a), 53 ± 6 nm for ethoxylated-SeNPs (Figure 1b), and 60 ± 6 nm for hydroxyethyl cellulose selenium nanoparticles (HEC-SeNPs) (Figure 1c). More than 1500 SeNPs dispersed in about 20 TEM photos were viewed to measure the size distribution. The results showed that the stabilizers employed provided spherical SeNPs of similar diameters, allowing us to examine the toxicity of SeNPs against the nature of the capping agent. Additionally, the electron diffraction pattern confirmed the non-microcrystalline structure of the synthesized SeNPs.

It is worthwhile mentioning that all the obtained SeNPs dispersions were stable at least for two months either in aqueous solution, or in a Man, Rogosa and Sharpe (MRS) broth media where the bacteria were cultured. NP stability is a key factor when evaluating toxicity effects. Aggregation of NPs can contribute to modify the effect of NPs on living systems when compared to non-aggregated NPs.

**Figure 2:**
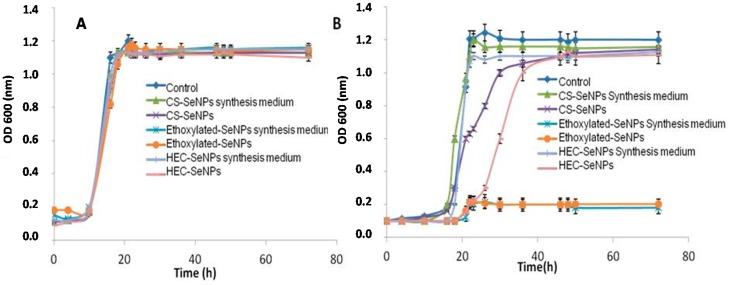
Effect of SeNPs and selenite on bacteria growth and accumulation. Bacteria growth curve in presence of SeNPs at two levels of exposure (**A**) 1 μg Se mL^−1^ and (**B**) 10 μg Se mL^−1^. Data are expressed as mean ± standard error *N* = 3 replicates.

**Figure 3:**
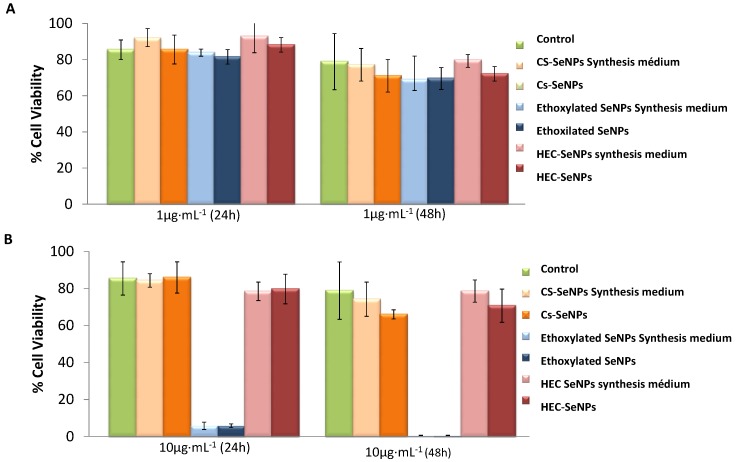
Percentages of cell viability of *L. d. bulgaricus* LB-12 after treatment with SeNPs at two level of exposure (**A**) 1 μg Se mL^−1^ and (**B**) 10 μg Se mL^−1^. Data are expressed as mean ± standard error *N* = 3 replicates.

The behavior of SeNPs on *L. d. bulgaricus* LB-12 was studied by evaluating bacteria growth by optical density at 600 nm (OD_600_) measurements (Figure 2) and bacteria viability using the LIVE/DEAD BacLightTM kit and flow cytometry analysis (Figure 3). Both assays were performed without (control) and with SeNPs at two concentration levels (1 and 10 μg Se mL^−1^). OD_600_ data collected in Figure 2 showed that bacteria growth was not affected by the presence of SeNPs when dosing 1 μg Se mL^−1^ of SeNPs (Figure 2A). Furthermore, the capping agents themselves did not show any effect on bacterial growth at this concentration level. However, bacterial growth rate declined when selenium level increased up to 10 μg Se mL^−1^. The influence was significant when bacteria were exposed to ethoxylated-SeNPs (Figure 2B). In fact, the presence of ethoxylated-SeNPs strongly inhibited bacterial growth. At this step, it is worth mentioning the role of the capping agents when working at this selenium concentration level. Chitosan and HEC did not have any impact on bacterial growth, suggesting that the bacterial growth decrease in the presence of SeNPs was due to the HEC-SeNPs or CS-SeNPs themselves, rather than the presence of impurities (reducing agent or stabilizer) in the SeNPs solution. However, the presence of the ethoxylated surfactant had a dramatic impact on bacterial growth and therefore in toxicity. As shown in Figure 2B, both the ethoxylated surfactant itself and the ethoxylated-SeNPs completely inhibited bacterial growth when compared to control samples. In this case, the toxicity of the SeNPs was clearly due to the capping agent. It is important to note that the 10 μg Se mL^−1^ ethoxylated-SeNPs solutions contains a higher concentration of capping agent than the 1 μg Se mL^−1^ of SeNPs solution. The results obtained by OD_600_ measurements were further confirmed by flow cytometry measurements. Figure 3 shows the number (as percentage) of bacterial cells stained with the green-fluorescent SYTO^®^ 9dye (SYTO 9) (intact cells) and with propidium iodine (PI) (damaged cells). Around 80% of bacteria cells were viable in the presence of 1 μg Se mL^−1^, after 24 and 48 h of incubation (Figure 3A). The observed behavior was independent of the type of SeNPs and the time of exposure. Additionally, the capping agents themselves did not affect bacterial cell viability. When a level of 10 μg Se mL^−1^ (Figure 3B) was supplemented, the presence of HEC-SeNPs or CS-SeNPs barely affected the viability of the exposed bacterial cells, even at an exposure time of 48 h. In contrast, the presence of 10 μg Se mL^−1^ of Se in form of ethoxylated-SeNPs during 24 and 48 h of exposure evidenced an important decrease in bacterial cell viability. These results reflected the importance of the nature of the capping agent in regards to bacterial cell viability.

Data reported in the current study demonstrated that several factors must be taken into consideration with regard to establish the true toxicity of SeNPs. First, the potential toxicity of the chemical reagent used as capping agent for stabilizing SeNPs suspensions. Concerning the toxicity observed when exposing *L. d. bulgaricus* LB-12 to ethoxylated-SeNPs, it has been clearly demonstrated that the toxic effects are mainly due to toxicity of the non-ionic surfactant. The results obtained are of importance since in most publications on NPs toxicity, the influence of the chemical reagent used in preparing NPs is not properly evaluated. It is worth mentioning that the stabilizer employed (2,4,7,9-tetramethyl-5-decyne-4,7-diol ethoxylate, called Surfynol 420) is a non-ionic surfactant which is commonly used in coatings related to different applications especially in food packaging. Our results shown that surfynol kills *L. d. bulgaricus* LB-12 cells being toxic at this concentration also for a short exposure time.

### 2.2. Bacteria Uptake and Transformation of SeNPs

**Figure 4:**
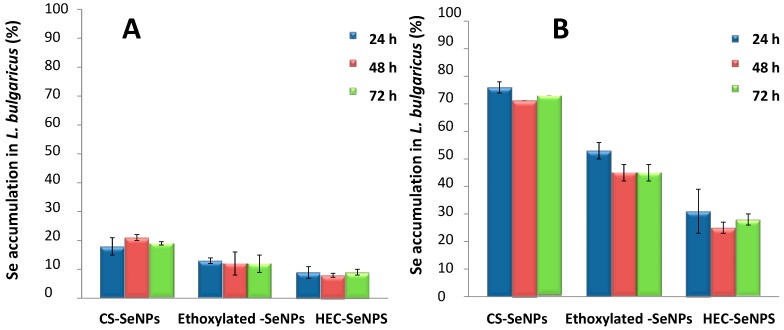
Selenium accumulation in bacteria in presence of SeNPs at two level of exposure (**A**) 1 μg Se mL^−1^ and (**B**) 10 μg Se mL^−1^ at different times of exposure. Data are expressed as mean ± standard error *N* = 3 replicates.

SeNPs accumulation by the bacteria along with transformation of SeNPs once accumulated for the bacteria are important data with the aim of getting understanding on the effect of SeNPs exposure. For this purpose, selenium concentration in both culture media and bacterial cell pellet of bacteria supplemented with 1 μg Se mL^−1^ and 10 μg Se mL^−1^ of SeNPs was determined by ICP-MS. As it is shown in Figure 4A, when dosing with 1 μg Se mL^−1^ accumulation low percentages of accumulation were achieved (10% and 20%). No statistically significant difference (α = 5%) was found between accumulation values obtained at several times of incubation (24, 48, 72 h, which corresponds to the stationary phase of the bacterial culture growth in Figure 2A) and with the three type of SeNPs. In contrast, selenium uptake increases when the selenium level of exposure (10 μg Se mL^−1^) increases with values of 80%, 50% and 30% for CS-SeNPs, for ethoxylated-SeNPs and for HEC-SeNPs, respectively (Figure 4B). Again, selenium accumulation seems to be independent of the time of exposure 24 (log phase in Figure 2B), 48 and 72 h (stationary phase of bacterial culture growth in Figure 2B). These results may suggest that selenium accumulation takes place during the log phase when bacteria growth occurs. The increase of Se concentration in the pellet was consistent with a progressive decrease in the culture media (data not shown).

**Figure 5:**
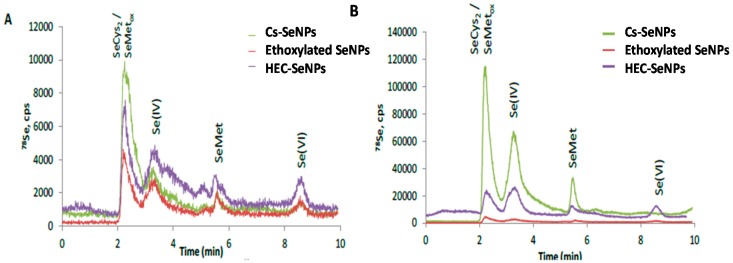
Transformation of SeNPs by *L. d. bulgaricus* LB-12. Anion-exchange high performance liquid chromatography on line coupled to inductively coupled plasma mass spectrometry (HPLC-ICP-MS) chromatogram of hydrolyzed extracts of bacterial cell pellet after exposure to (**A**) 1 μg Se mL^−1^ and (**B**) 10 μg Se mL^−1^ over 24 h. SeMet: Selenomethionine, SeMetox: Selenonmethionine-Se-oxide and Se(IV) selenite.

**Figure 6:**
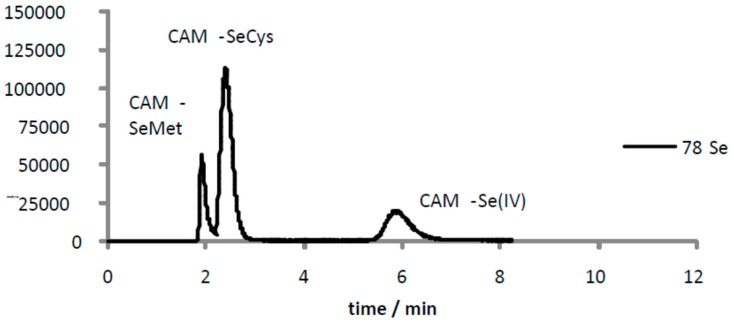
Anion exchange HPLC-ICP-MS chromatograms of hydrolyzed extracts of CS-SeNPs bacterial cell pellet after exposure to 10 μg Se mL^−1^ as Cs-SeNPs obtained by carbamidomethylation following by enzymatic hydrolysis. CAM-SeCys: carbomethylated selenocysteine; CAM-SeMet: carbomethylated selenomethionine; and CAM-Se(IV): carbomethylated selenite. Measurements were performed by monitoring ^78^Se isotope.

Selenium species in the bacteria pellet and culture medium was measured by anion-exchange chromatography on line coupled to inductively coupled plasma-mass spectrometry (ICPMS). For this purpose, selenium species were first enzymatically hydrolyzed using a mixture of enzymes consisting of lysozyme and protease in a buffered media at pH = 7.0, composed of 40 mM TRIS-HCl, 20 mM acetic acid and 1mM EDTA and commonly named as (TAE). Lysozyme was selected since it is able to break the bacterial cell wall allowing entry of protease into the bacteria. Recovery values in extracting selenium species were approximately 100%. Subsequently, the extracts were measured by high performance liquid chromatography on line coupled to inductively coupled plasma mass spectrometry (HPLC-ICP-MS). Selenium was quantitatively recovered (100 ± 4) from the injection of selenium species standard. Figure 5 shows the chromatographic profiles derived from bacteria exposed to three different SeNPs solutions over 24 h. Selenocystine (SeCys_2_), selenomethionine (SeMet) and inorganic selenium were the major selenium species identified by comparing the retention time of the standards and by spiking experiments. One of the main problems when identifying selenocysteine (SeCys) by anion exchange-chromatography is that this selenocompound eluted at the void volume as a non-retained compound. Moreover, selenocysteine residue decomposition during sample preparation may occur. To overcome these problems, SeCys residues were alkylated with iodoacetamide prior to enzymatic hydrolysis [18] Extracts were analyzed by HPLC-ICP-MS. The anion-exchange chromatogram in Figure 6 shows a Se-containing peak at 3 min that was identified as the carbomethylated derivative of SeCys. The same protocol was applied for ethoxylated-SeNPs and HEC-SeNPs treated bacteria. This procedure allowed us to perform unambiguous assignment of the Se-compounds. The results obtained agreed well with data previously reported and evidence the ability of *L. d. bulgaricus* LB-12 to biotransform inorganic selenium into selenium organic compounds [16,17,18,19,20]. Selenium species were further quantified by HPLC-ICP-MS by applying the standard addition method. Differences in selenium species distribution was observed, depending on the SeNP solution added to the culture media, and the level of exposure. In bacteria exposed to 1 μg Se mL^−1^ as CS-SeNPs, ethoxylated-SeNPs and HEC-SeNPs, around 95%, 70% and 90% of the total selenium accumulated respectively in the bacterial cell pellet was transformed into Se organic species, and mainly as SeCys and SeMet. In contrast, when bacteria were exposed to 10 μg Se mL^−1^ of CS-SeNPs, ethoxylated-SeNPs and HEC-SeNPs, around 90%, 10% and 35% of the selenium was found to be present as organic forms, respectively. It is important to mention that SeCys was the major selenium specie found when treating bacteria with CS-SeNPs. In contrast, the presence of 10 μg Se mL^−1^ of ethoxylated-coated SeNPs in the culture media strongly inhibited the metabolic synthesis of organoselenium compounds, with selenium mostly accumulating as inorganic selenium (90%). These results are consistent with the dramatic decreases in viability observed in ethoxylated-coated SeNP-exposed cells compared to control cells.

The difference in SeNPs transformation observed among the SeNPs solution tested may be attributed to differences in bacteria uptake metabolisms. Two basic mechanisms of ions binding have been described in bacteria: bioadsorption and bioaccumulation [21]. To gain a deeper insight into the SeNPs uptake mechanisms, the degree of SeNPs internalization was evaluated by TEM after fixing bacteria following the experimental procedure described in Section 3.4.

### 2.3. Localization of SeNPs by Transmission Electron Microscopy (TEM)

**Figure 7:**
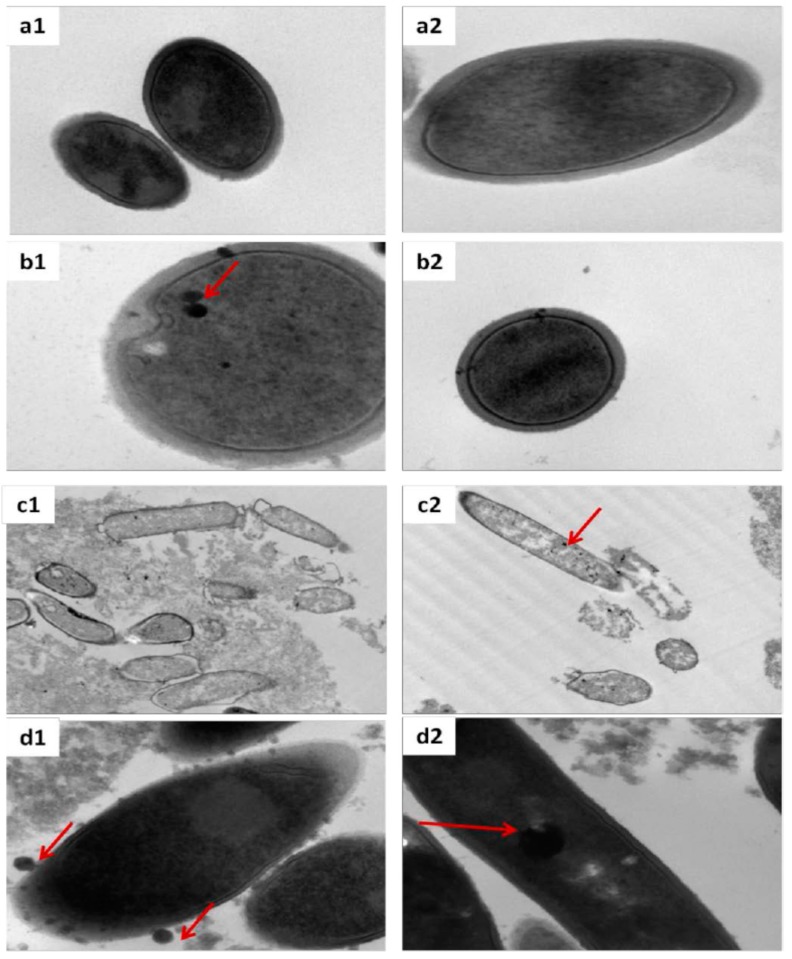
Transmission electron micrographs of un-exposed bacteria (**a1**,**a2**) and exposed bacteria treated with 10 μg·mL^−1^ of Se for 48 h, as CS-SeNPs (**b1**,**b2**), ethoxylated-SeNPs (**c1**,**c2**) and HEC-SeNPs (**d1**,**d2**). Red arrows indicate the presence of nanoparticles.

The degree of NP internalization is an important parameter when evaluating the effect of SeNPs exposure in biological specimens. In the current study, TEM was used to locate SeNPs in the bacteria cells with the aim of determining whether SeNPs enter inside the bacteria or on the contrary, are just attached to bacteria cell wall. Figure 7 displays TEM micrographs from control and selenium-exposed bacteria. Only TEM micrographs of bacteria supplemented with 10 μg Se mL^−1^ of SeNPs are depicted because no differences in bacteria morphology were observed when they were exposed to 1 μg Se mL^−1^ of SeNPs.

Figure 7(a1,a2) displays the characteristic bacilli shape of an unexposed *L. bacillus* with a cell wall of uniform thickness (28 to 35 nm) with an electron dense outer layer and an inner layer resembling unit-membrane structure. Concerning CS-SeNPs treated bacteria, Figure 7(b1,b2) suggests that CS-SeNPs are able to enter inside the cell causing a limited cellular damage by keeping the bacteria cell wall intact. These results agreed well with previous results derived from the viability assays. In contrast, bacteria cells exposed to HEC-SeNPs and Ethoxylated-SeNPs displayed the most significant ultrastructural changes, including disruption of intracellular components. *L. bulgaricus* exposed to HEC-SeNPs Figure 7(d1,d2) displayed cytoplasm condensation while keeping cell wall integrity. As it is shown, HEC-SeNPs were mostly attached to the cell wall. Only a few HEC-SeNPs may have been able to pass through the bacteria cell wall to be metabolically transformed into organo-Se-compounds, explaining the low amount of Se-organocompounds found (35%). In contrast, the toxic effect of non-ionic surfactant on bacteria is highly evident in Figure 7(c1,c2). TEM micrographs show that, under these conditions, the cell wall was completely disrupted in most of the exposed bacteria, leading to bacteria cell death, corroborating both the data from viability assay and the poor rate of ethoxylated-SeNP biotransformation to organoselenocompounds (10%) in the cell wall.

It appears that the interaction of SeNPs with *L. d. bulgaricus* LB-12 depended on the capping agent used, and a combination of both physical and chemical interaction may have taken place. The results obtained suggest that biosorption has an important effect on the accumulation of ethoxylated-SeNPs and HEC-SeNPs by the bacteria. NPs are first adsorbed onto the cell wall and then interact with the cellular membrane, either without compromising membrane integrity (as HEC-SeNPs) or causing physical damage, consequently leading to bacteria death (as ethoxylated-SeNPs). SeNPs are also partially dissolved and non-metabolically transformed into inorganic selenium with the assistance of substances excreted by the bacteria cell wall. Moreover, transformation of SeNPs may also affect the capping agent. The capping agent may be desorbed by the bacteria and may greatly affect the toxicity of SeNPs, this being a process of special relevance when applying ethoxylated-SeNPs. The data in the current study suggested that the true toxicity of ethoxylated-SeNPs was mainly due to the capping agent rather than SeNPs themselves.

In contrast, CS-SeNPs are bioaccumulated inside the bacterial cells. CS-SeNPs first attach to the bacteria cell wall and then pass through the membrane without causing damage. Once inside, SeNPs are metabolically transformed to selenocompounds. This fact could explain the results obtained in bacteria cell viability.

## 3. Materials and Methods

### 3.1. Chemicals

Chemicals employed in the current study were of analytical grade. De-ionized water (18 M·Ω·cm) from a Milli-Q water purification system unit (Millipore, Belford, MA USA) was used for preparing solutions. SeMet, MeSeCys and SeCys_2_ were purchased from Sigma (Sigma Chemicals, St. Louis, MO, USA) and their respective standard solutions were made by dissolving in 3% hydrochloric acid (37%, Merck, Darmstad, Germany). Inorganic selenium solutions were obtained from sodium selenite (Na_2_SeO_3_) and selenate (Na_2_SeO_4_) salts acquired from Merck. 1000 mg·L^−1^ stocks solutions were maintained at 4 °C and working solutions were prepared by dilution when needed. The capping agents employed for synthesis were the polysaccharide Chitosan, derived from shrimp shells (340 g·mol^−1^·MW and ≥75% deacetylation degree); a non-ionic surfactant: 2,4,7,9-tetramethyl-5-decyne-4,7-diol ethoxylate from Sigma (Sigma Chemicals, St. Louis, MO, USA) and a polymer such as hydroxyethyl cellulose from Sigma (Sigma Chemicals, St. Louis, MO, USA). Ascorbic acid was obtained from Sigma (Sigma Chemicals, St. Louis, MO, USA).

Total selenium concentration in SeNP-treated bacteria pellets and culture media was determined by acid digestion of samples using HNO_3_ (Merck) and 30% hydrogen peroxide (Panreac, Barcelona, Spain). SeNPs-treated bacteria pellet underwent to enzymatic hydrolysis for extraction of selenium species. For this purpose, Protease XIV and lysozyme from Sigma in presence of TAE Buffer, (consisting of 40 mM Tris, 20 mM Acetic Acid, 1 mM EDTA at pH 7.0) were used. Selenium species analysis was achieved by anionic-exchange chromatography coupled to ICPMS. A solution containing 10 mM citric acid (Sigma) in 2% methanol (MeOH, 99.9%, Scharlab S.L., Barcelona, Spain) at pH 5 was employed as the mobile phase.

Bacteria membrane integrity was determined by employing the fluorescent dyes SYTO 9 and PI of the LIVE/DEAD^®^ BacLight™ bacterial viability kit (Molecular Probes, Invitrogen, Thermo-Fisher Spain, Madrid, Spain).

### 3.2. Synthesis of Selenium Nanoparticles

SeNP synthesis was achieved by applying a chemical procedure described by Bai et al. [11] based on the reduction of selenite with ascorbic acid. Three different types of SeNP were obtained and employed in present work: (1) chitosan, (CS-SeNPs); (2) 2,4,7,9-tetramethyl-5-decyne-4,7-diol ethoxylate coated SeNPs (Ethoxylated-SeNPs) and (3) Hydroxyethyl cellulose; HEC-SeNPs. The selected conditions for obtaining spherical SeNPs with a formation yield around 95% were: 0.054 M ascorbic acid, 1000 μg·mL^−1^ of selenium as selenite and a stabilizer agent concentration of 0.1% at pH 5. The excess of reagents used for SeNPs synthesis were removed by means of a dialysis process using a dialysis membrane with a molecular mass cut-off of 12 kDa. The resulting aqueous suspensions were diluted to a final concentration of 200 mg·L^−1^ of SeNPs. SeNPs remain stable at 4 °C for two months. 

### 3.3. Bacterial Culture and Nanoparticle Application

The strain employed was *Lactobacillus delbrueckii* subsp. *bulgaricus* LB-12, which was obtained from F-DVS, Chr. Hansen’s Laboratory (Milwaukee, WI, USA). The bacterial culture was grown in MRS broth (Scharlab S.L., Barcelona, Spain) according to the manufacturer’s instructions. The cultures supplemented with 1 μg Se mL^−1^ and 10 μg Se mL^−1^ as CS-SeNPs, Ethoxylated-SeNPs and HEC-SeNPs were incubated at 37 °C during 24, 48 and 72 h, and subsequently centrifuged at 5000 rpm for 5 min at 4 °C and washed twice with 1 mL Milli-Q water. Both bacteria pellet and culture media were stored at −80 °C until analysis. *L. d. bulgaricus* LB-12 without adding SeNPs was growth in parallel as control assay.

### 3.4. Characterization of SeNPs Suspensions and SeNPs Internalization in Bacteria by TEM

SeNPs were characterized in terms of size and morphology by high resolution transmission electron microscope JEM 2100 (JEOL, Peabody, MA USA) with an X-ray energy dispersive spectroscopy (XEDS) microanalysis composition system (Oxford Instruments, Oxford, UK). Samples were first treated by evaporating a drop of SeNP dispersion onto a 300 mesh lace carbon copper grid.

The degree of internalization of SeNPs in bacteria was measured by TEM after fixing the bacteria in situ with glutaraldehyde (2.5% *v/v*) and p-formaldehyde (4% *v/v*) in PBS at 4 °C for 4 h. Afterwards, lactic bacteria were washed and stored in PBS at 4 °C overnight. Bacteria were treated with osmium tetroxide (1% *v/v*) over one hour at room temperature in darkness. Subsequently, bacteria were dehydrated in graded ethanol series (from 30 to 100% ethanol) and harvested with propylene oxide. The pellets were treated with a mixture of resin:acetone and finally, treated with 100% resin. The resulting blocks were submitted at 65 °C during 48 h. Sections were cut and placed onto copper grids for TEM measurements.

### 3.5. Selenium Analysis

#### 3.5.1. Total Selenium Determination in Bacterial Cell Pellet and Culture Medium

About 100 mg of SeNP-treated bacterial cell pellet and/or 0.5 mL of culture medium were submitted to acid digestion by adding 1 mL of concentrated HNO_3_ and 0.5 mL of 30% H_2_O_2_. Acid digestion was performed in a 1000 W MSP microwave oven (CEM, Matthews, NC, USA). The obtained digests were diluted with Milli-Q water. Selenium concentration was determined by ICPMS using an Agilent7700-collision/reaction cell ICP-MS (using H_2_ collision gas) instrument and by applying the experimental conditions detailed in Table 1.

#### 3.5.2. Selenium Species Determination in Bacterial Cell Pellet

Selenium species determination in the SeNPs-treated bacterial cell pellet was achieved by using a two-step enzymatic hydrolysis protocol. First, 500 μL of 100 μg·mL^−1^ of lysozyme (Sigma Aldrich, Steinheim, Germany) solution in TAE Buffer (pH 7.0; Tris 40 mM, Acetic acid 20 mM, EDTA 1 mM) were added to 0.050 mg of bacterial cell pellet; the mixture was incubated for 2.5 h at 37 °C. Subsequently, enzymatic hydrolysis by adding 500 μL of 100 μg·mL^−1^ protease type XIV (Sigma Aldrich, Steinheim, Germany) solution in TAE buffer was performed. With the aim of decreasing treatment time, enzymatic hydrolysis was carried out by means of using an ultrasonic probe (Sonoplus ultrasonic with a 3 mm diameter titanium microtip, Bandenlin, Berlin, Germany) during 50 s at 60% of ultrasound amplitude. Selenium species in the culture media were enzymatically hydrolyzed by adding 500 μL of 100 μg·mL^−1^ protease type XIV in TAE buffer followed by 50 s of tip-sonication. The extracts were centrifuged at 7500× *g* during 15 min using 0.22 μm cutoff-filter and storage to −80 °C until analysis. Selenocysteine integrity was maintained by applying a method previously employed by the authors [18] and consisted on the use of urea, then dithiotheitol (DTT) to conduct the reduction of Se–S, Se–Se bridges and finally alkylation with iodoacetamide. For this purpose, the protein soluble fraction from the bacteria pellet was treated with 2 mL of 7 M urea in 0.1 M Tris pH 7.5. Afterwards, samples were shaken and kept in the dark with 0.2 M DTT in 0.1 M Tris and 0.5 M iodoacetamide for 1 h at each step. The excess of iodoacetamide was eliminated by adding 375 μL of DDT. Once the reaction was completed, the resulting carbomethylated samples were diluted with Milli-Q water and underwent enzymatic hydrolysis following the procedure described above.

Anion exchange chromatography (Hamilton PRP X-100, 150 mm × 4.6 mm, 10 μm, Hamilton, Switzerland) on line coupled to ICPMS was used to determine selenium species in the extracts. Selenium compounds recovery on the column was evaluated by introducing the selenium species standards in the ICPMS by means of using a flow injection system. For each selenium species, peak areas obtained by flow injection set-up were compared with those peaks areas obtained in the chromatogram. Selenium species were quantified by applying the standard addition method. Measurements were carried out by selecting ^76^Se, ^77^Se, ^78^Se, and ^80^Se isotopes.

### 3.6. Flow Cytometry Assessment of Viability of Lactic Bacteria Treated With SeNPs

The effect of SeNPs *on L. d. bulgaricus LB-12* viability was measured by employing the fluorescent dyes of the LIVE/DEAD^®^ BacLightTM bacterial viability kit. *L. d. bulgaricus* LB-12 were treated with 1 μg Se mL^−1^ and 10 μg Se mL^−1^ of Se (in form CS-SeNPs, HEC-SeNPs and Ethoxilated-SeNPs) for 24 and 48 h, and then centrifuged. After exposure, bacterial cells were washed, resuspended and diluted with NaCl (0.85% *w/v*) to get a concentration of 1 × 10^6^ bacteria mL^−1^ in 1 mL NaCl solution. Subsequently, 1.5 μL of each dye (3.34 mM of SYTO 9 dye and 20 mM propidium iodide) were spiked to 1000 μL of this diluted bacterial cell suspension. Additionally, aliquots of 1000 μL of diluted bacterial cell suspension were also treated with SYTO 9 alone and PI alone. The resulting suspensions were kept for 15 min at room temperature in darkness and immediately measured by cytometry analysis. A fluorescence-active cell sorting (FACS) Calibur flow cytometer (Becton Dickinson Immunocytometry Systems, San Jose, CA, USA) with a 15 mW, 488 nm, air-cooled argon ion laser and a cell-sorting catcher tub was employed for assessing viability.

## 4. Conclusions

The results presented in this paper evidence the importance of the capping agent on SeNP behavior. The mechanisms of interaction may combine physical and chemical processes. TEM micrographs of *L. d. bulgaricus* LB-12 exposed to CS-SeNPs have shown that CS-SeNPs are able to enter inside the cell, causing a limited cellular damage by keeping the bacteria cell wall integrity intact. Moreover, around 90% of the selenium content found in CS-SeNPs-treated bacteria was metabolically transformed to organic species. Unlike CS-SeNPs, a dramatic decrease in *L. d. bulgaricus* LB-12 viability was observed when applied ethoxylated SeNPs. The data obtained suggests that the toxicity of ethoxylated-coated SeNPs is mainly governed by the toxicity of the surfactant used as capping agent. Consequently, the capping agent appears to play a key role to be considered when accurately assessing the toxicological and environmental behavior of SeNPs. However, the effect of the nature of capping agent is not mentioned in most of the studies appeared in the literature.

## Figures and Tables

**Table 1:** Operating conditions for high performance liquid chromatography (HPLC) coupled to inductively coupled plasma-mass spectrometry (ICPMS).

**Operating Conditions**	
**ICPMS Parameters for Se Determination**	
Radiofrecuency (RF) power (W)	1550
Plasma gas flow rate (L min^−1^)	15.0
Ar auxiliar flow rate (L min^−1^)	0.30
Carrier gas flow rate (L min^−1^)	0.75
Nebulizer	Slurry
Spray Chamber	Scott
Adquisition mode	Continuos
Isotopes monitored	^76^Se, ^77^Se, ^78^Se, ^80^Se
Replicates	3
Reaction gas	H_2_
Reaction gas (mLH_2_ min^−1^)	6
**High Performance Liquid Chromatography Parameters**	
Column	Hamilton PRP X-100 (150 mm × 4.6 mm, 10 μm)
Mobile phases	Ammonium citrate 10 mM, 2% MeOH (pH 5.0)
Mode	Isocratic
Flow rate (mL min^−1^)	1
Injection volume (μL)	100
